# Association between body energy content in the dry period and post-calving production disease status in dairy cattle

**DOI:** 10.1017/S1751731117000040

**Published:** 2017-02-15

**Authors:** G. L. Smith, N. C. Friggens, C. J. Ashworth, M. G. G. Chagunda

**Affiliations:** 1 Future Farming Systems, Scotland’s Rural College, West Mains Road, Edinburgh EH9 3JG, Scotland, UK; 2 INRA UMR 0791 Modélisation Systémique Appliquée aux Ruminants, INRA, AgroParisTech, Université Paris-Saclay, Paris 75005, France; 3 Division of Developmental Biology, The Roslin Institute and the R(D)SVS, University of Edinburgh, Edinburgh EH25 9RG, UK

**Keywords:** transition period, lactation management, production disease, disease indicators

## Abstract

The transition from gestation to lactation is marked by significant physiological changes for the individual cow such that disease incidence is highest in early lactation. Around the time of calving, cows rely on mobilisation of body energy reserves to fill the energy deficit created by an increase in nutrient demands at a time of restricted feed intake. It is well established that monitoring of body energy reserves in lactation is an important component of herd health management. However, despite their influence on future health and productivity, monitoring of body energy reserves in the dry period is often sparse. Further, there is increasing concern that current dry off management is inappropriate for modern cattle and may influence future disease risk. This study aimed to identify candidate indicators of early lactation production disease from body energy data collected in the dry period and production data recorded at the time of dry off. Retrospective analysis was performed on 482 cow-lactations collected from a long-term Holstein-Friesian genetic and management systems project, the Langhill herd in Scotland. Cow-lactations were assigned to one of four health groups based on health status in the first 30 days of lactation. These four groups were as follows: healthy, reproductive tract disorders (retained placenta and metritis), subclinical mastitis and metabolic disorders (ketosis, hypocalcaemia, hypomagnesaemia and left displaced abomasum). ANOVA, employing a GLM was used to determine effects for the candidate indicator traits. Cows which were diagnosed with a reproductive tract disorder in the first 30 days of lactation experienced a significantly greater loss in body energy content, body condition score and weight in the preceding dry period than healthy cows. The rate of change in body energy content during the first 15 days of the dry period was −18.26 MJ/day for cows which developed reproductive tract disorder compared with +0.63 MJ/day for healthy cows. Cows diagnosed with subclinical mastitis in the first 30 days of lactation had significantly greater milk yield at dry off in the previous lactation than cows that developed a reproductive tract disorder or metabolic disease in addition to a significantly higher yield to body energy content ratio at dry off than healthy cows. Physiological and production traits recorded in the lactation and dry period preceding a disease event differed between cows which developed different diseases post-calving. Differences in these traits allow the development of new disease indicators for use in models for the prediction of disease risk in the transition period.

## Implications

The importance of transition cow management has been well documented for some time. However, traditionally the transition period is considered to extend only 30 days each side of calving. Further, the assessment of body energy reserves by body condition scoring (BCS) is mostly conducted during lactation. We hypothesise that monitoring of energy reserves from the end of lactation and throughout the dry period would help mitigate early lactation disease. This paper describes traits which have potential as disease indicators in early lactation, sourced from data recorded in the lactation and dry period.

## Introduction

Production disease in early lactation poses a threat to animal welfare and the economic viability of dairy production. Diseases in early lactation account for a considerable proportion of health control costs in dairy farming systems, both directly and indirectly (Fourichon *et al*., 2001). Direct costs include the cost of veterinary treatment, whereas indirect costs are incurred through reduced fertility and longevity of affected cows. In 2014, the cost per case of left displaced abomasum and retained placenta were estimated to be £255 and £378, respectively (Cattle Health and Welfare Group, 2014).

The transition period, traditionally defined as extending from 3 weeks prior until 3 weeks post-calving, represents a significant physiological challenge for dairy cattle. During this time cows must adapt to the demands of lactation, while delivering healthy offspring, in the face of reduced feed intake, negative energy balance (NEB), insulin resistance and reduced immune function (Loor, 2013). Such is the challenge of the transition period that early lactation is marked by the highest disease incidence of any stage in the lactation–gestation cycle (Ingvartsen *et al*., 2003). It is estimated that 30% to 50% of cows are affected by some form of metabolic or infectious disease around calving. Disease associated with the early lactation period can be considered as ‘production disease’ and includes diseases which are induced and exacerbated by nutrition and management practices (Markusfeld, 2003). The abrupt cessation of milking at the time of dry off is an example of a widely practiced end of lactation management strategy which can have a significant effect on cow health. It has recently been suggested that this sudden cessation of milk removal causes discomfort and distress to the cow (Zobel *et al*., 2015). In addition, sudden dietary changes often occur between the lactating and dry periods. The rumen environment must adapt to a change from an energy dense lactation diet to one which meets basic maintenance requirements, before preparation begins in the transition period to adjust back to the lactation ration (Dingwell et al., 2001). Concerns have been raised that such a major change in nutrient supply at dry off may lead to metabolic disorders in the transition period and ensuing lactation, especially among high-yielding cows (Odensten *et al*., 2007).

Assessment of body energy reserves using BCS is one strategy which can be employed to monitor transition cow management. Body energy content of individual cows is dependent on energy intake, energy output and energy reserves retained from previous lactation stages (Banos *et al*., 2006). In early lactation, energy output for milk production far exceeds energy intake and thus requires the mobilisation of body energy reserves to meet the energy deficit. Although energy balance, the change in body energy stores, is normally monitored closely in early lactation cows, monitoring of body energy status in the dry period is sparse (Rutten *et al*., 2013). Further, to solely focus on the transition period may mean that vital disease indicators from the end of lactation and throughout the dry period may be missed or excluded.

The hypotheses of this study were that both (a) differences in body energy content (BEC) traits measured over the dry period and (b) differences in physiological and production traits recorded during the changeover period exist between cows that develop different production diseases in the first 30 days of lactation. The changeover period refers to the period in which the switch from a lactating to dry state is made. Therefore, the objective of this study was to determine the association between BEC in the dry period and post-calving production disease status. This was performed with a view to identification of candidate indicators of disease, recorded in the changeover and dry periods, which could be used to distinguish between healthy cows (HC) and non-HC.

## Material and methods

Data were collected over an 8-year period (November 2003–September 2011) during the long-term genetic by environment study at Scotland’s Rural College (SRUC) Dairy Research and Innovation Centre, Crichton Royal Farm, Dumfries, Scotland. A total of 482 cow-lactations from 399 multiparous cows were analysed. Data from primiparous animals were not included due to differences in physiology and management.

### Experimental design and animals

Experimental design of the long-term study has previously been described in detail by Pryce *et al*., 1999). In short, two genetic lines of Holstein-Friesian cattle were selected to represent average UK genetic merit for milk fat and protein (control) and the top 5% of UK genetic merit for the trait (select). Within each of the genetic lines, cows were assigned to one of two dietary treatments – high forage or low forage. Dietary treatments were described in detail by Chagunda *et al*. (2009). In short, high forage cows were grazed when grass growth permitted and fed a complete diet containing 70% to 75% forage, on a dry matter (DM) basis, when housed. Cows in the low forage system were housed continuously and fed a complete diet of 40% to 45% forage on a DM basis. All herd groups were subject to the same procedures with respect to health and fertility management. Cows were dried off at ~7 months gestation and treated with a long acting intramammary antibiotic. In the far-off dry period (from dry off until 3 weeks before predicted calving date) cows were housed in cubicles and fed a straw-based ration. The diet comprised 45% straw, supplemented with grass and maize silages, whole-crop wheat silage, concentrate blend, soya and minerals. Cows were moved to straw pens 3 weeks before predicted calving date and fed a transition diet which consisted of one-third of the lactation ration for their respective production group (i.e. low forage or high forage) supplemented with straw.

### Data recording

Cows were milked three times daily and milk yield (MY) was recorded at each milking. Proportional milk samples were taken once weekly and analysed for fat, protein and somatic cell count. Body weight was measured three times daily on exit from the milking parlour by means of a walk over weigh scale (Insentec BC, Marknesse, The Netherlands). Body condition score was assessed and recorded weekly throughout the lactation and dry periods by trained assessors following standardised protocols using a 0 to 5 scale as per Lowman *et al*. (1973). Assessors alternated weekly to reduce the effect of operator bias and regular re-training was provided.

All disease diagnoses were performed by either a veterinarian or a senior stockperson and recorded in the herd database. Standard operating procedures for the identification of diseases were in place throughout the study period to ensure consistency and to reduce human bias. Senior staff were responsible for diagnosing cases of lameness, subclinical mastitis (SCM) and clinical mastitis and retained placenta. Suspected cases of metritis, ketosis, hypocalcaemia, hypomagnesaemia and left displaced abomasum were identified by stock workers before formal diagnosis by a veterinarian. All data were held and managed in a SQL database. Analysis was performed in SAS v9.3.

### Data handling

#### Classification of cow-lactations

Cow-lactations were assigned to one of four groups based on disease incidence in the first 30 days of lactation. These groups were HC, reproductive tract disorder (REP), SCM and metabolic (MET). Cow-lactations with clinical mastitis were not included in the analysis. Definitions for each of these groups are outlined in Table 1.

**Table 1:** Criteria used to classify cow-lactations by health group

Health groups	Definition
Healthy cows (HC)	No clinical disease diagnosis and somatic cell count number >250 000 cells/ml in the first 30 days of lactation
Subclinical mastitis	At least one recorded somatic cell count >250 000 cells/ml in the first 30 days of lactation
Reproductive tract disorders	Clinical cases of metritis (abnormally enlarged uterus, vaginal discharge and systemic illness/fever with a temperature >102.5°F) and retained placenta (failure to expel foetal membranes within 6 h of calving) – diagnosed by veterinarian in the first 30 days of lactation
Metabolic disorders	Clinical cases of hypocalcaemia (low blood calcium levels, lack of rumen activity and recumbency), hypomagnesaemia (low blood magnesium levels, excitability/hypomagnesaemic tetany), left displaced abomasum (sudden decrease in milk yield, reduced feed intake secondary ketosis) and ketosis (decreased concentrate intake, lethargy and abnormal behaviour) – all diagnoses confirmed by veterinarian in the first 30 days of lactation

Low incidence rates for hypocalcaemia, hypomagnesaemia, left displaced abomasum and ketosis necessitated their combination to form the ‘MET’ group. Cows diagnosed with multiple diseases, which accounted for 0.2% of the cow-lactation records, used in this study, were assigned to the health group of the most severe health event. For the purposes of this study metabolic diseases were categorised as the most severe, followed by REP and then SCM. Metabolic disorders were categorised as the most severe due to their systemic nature and their long lasting effects on health and productivity (Stangaferro *et al*., 2016). Clinical incidences of REPs were classified as more severe than SCM. Classification of cow-lactations by production system and parity are given in Table 2.

**Table 2:** Health group classifications in early lactation by production system and parity for 482 cow-lactations from the Holstein-Friesian dairy herd, Crichton Royal Farm, Scotland’s Rural College Dairy Research and Innovation centre (November 2003 to September 2011)

	Health classification				
	Healthy cows (*n*)	Subclinical mastitis (*n*)	Reproductive track disorders^1^ (*n*)	Metabolic disorders^2^ (*n*)	
Production system					
Low forage control^3^	93	14	20	4	
Low forage select^4^	63	16	19	3	
High forage control^3^	106	13	19	3	
High forage select^4^	73	10	19	7	
Parity					
2	203	25	42	5	
3	132	28	35	12	
Total	335	53	77	17	482

1 Includes cases of retained placenta and metritis.

2 Includes cases of left displaced abomasum, hypocalcaemia, hypomagnesaemia and ketosis.

3 Control cows were selected to represent average UK genetic merit for milk fat and protein.

4 Select cows were selected to represent the top 5% of UK genetic merit for milk fat and protein.

#### Calculation of candidate indicator traits

Body energy content was calculated using standard equations using weekly BW and BCS data for each week of the dry period (Banos *et al*., 2006). The arithmetical difference in BW, BCS and BEC between dry off and calving were calculated for each cow-lactation. The rate of change in BW, BCS and BEC during the first 15 days of the dry period was calculated by fitting a regression model to recorded data for each trait. The ratio of daily energy-corrected milk (ECM) yield to daily BEC was calculated as ECM (L) on the day of dry off per 100 MJ of the cows BEC on the day of dry off (MBER). This milk yield-to-body energy ratio (MBER), represented the propensity of an individual cow to sustain high MYs at the end of the lactation, whereas maintaining high body condition to support milk production and the growing foetus (Wathes *et al*., 2007). Milk yield was converted to ECM, using the method by Sjaunja *et al*. (1990).

### Statistical analysis

ANOVA, employing a GLM was used to determine effects for the candidate indicator traits. The model included fixed effects of health group, production system, dry period length, parity and calendar year. Cow-lactation was included as a random effect. Calendar year was included to account for year-to-year variations in weather and feed resources over the 8-year-study period. The same model was used to analyse each trait which were treated in turn as outcome variables. The model statement also initially included calf weight and sex, but these were later removed from the model as they were found not to influence the measured variable. Further, calf weight was correlated with dry period length. Significant differences between variables were determined by pair wise comparisons using the Tukey method. Data were analysed using the GLM procedure of SAS (SAS software version 9.3; SAS Institute Inc., Cary, NC, USA).

## Results

### Effects of production system and parity on dry period traits

There were significant effects of production system on all traits (BW, BCS and BEC) (*P*<0.05) (Table 3). Low forage cows had significantly greater BEC at drying (*P*<0.05) and incurred a loss in BEC of more than double that of high forage cows across the dry period. During the first 2 weeks of the dry period high forage control cows gained 9.97 MJ/day, whereas low forage select cows lost 13.7 MJ/day. BCS was significantly lower in high forage select cows at the time of drying than in all other groups (*P*<0.01). At calving, a significant difference in BCS existed between cows from the low forage control group and the high forage select group (*P*<0.01). The difference in BCS between dry off and calving was significantly different between cows of different production systems (*P*<0.001). Low forage control cows lost more than double that of cows from both high forage groups. Body weight was similarly significantly different between cows from different production systems. At dry off cows in the high forage control system were significantly (*P*<0.001) lighter than cows from all other systems. They remained the lightest throughout the dry period however, their weight was not significantly different to cows from the low forage system at calving. Cows fed a low forage diet, irrespective of genetic merit, had a negative slope of change in BW during the first 15 days of the dry period. However, the only significant difference which existed among the systems was between low forage control and high forage control cows (*P*<0.001). Throughout the dry period, cows in the low forage control group lost significantly more BW (57.5 kg) than cows in either of the high forage systems (*P*<0.001). There were significant effects of parity on BEC and BW at drying and calving, with parity 3 cows having significantly higher average BEC at BW than those in parity 2 (*P*<0.001). Parity 3 cows lost significantly (*P*<0.001) more BEC over the dry period than cows in parity 2. Body condition score was significantly higher at dry off in parity 3 cows (*P*<0.01). In the first 15 days of the dry period, parity 3 cows gained on average 0.82 kg/day, whereas parity 2 cows lost 0.58 kg/day.

**Table 3:** Least squares means and associated SEs of the effect of production systems and parity on body energy content, body condition score and BW at drying, calving, the rate of change in the first 15 days of the dry period and the arithmetical difference in the traits between drying and calving in Holstein dairy cattle

	Production system									Parity				
	Low forage control		Low forage select		High forage control		High forage select			2		3		
	Mean	SE	Mean	SE	Mean	SE	Mean	SE	*P*	Mean	SE	Mean	SE	*P*
Body energy content														
Drying (MJ)^1^	3443^a^	133	3340^a^	141	2948^b^	136	2808^b^	138	*	2929^B^	119	3353^A^	120	***
Calving (MJ)^2^	2818^ab^	104	2852^a^	109	2673^ab^	106	2602^b^	108	*	2586^B^	93	2887^A^	97	***
Difference (MJ)^3^	−612^a^	107	−493^a^	112	−222^b^	108	−149^b^	110	**	−311^A^	98	−427^B^	98	***
Slope (MJ/day)^4^	−7.44^ac^	7.28	−13.7^a^	7.58	9.97^b^	7.38	6.25^bc^	7.50	**	−3.51	6.57	1.03	6.57	Ns
Body condition score														
Drying^1^	2.53^a^	0.06	2.44^ab^	0.06	2.34^b^	0.06	2.19^c^	0.06	**	2.34^b^	0.06	2.42^a^	0.06	**
Calving^2^	2.26^a^	0.05	2.19^ab^	0.05	2.22^ab^	0.05	2.09^b^	0.05	**	2.17	0.04	2.21	0.04	Ns
Difference^3^	−0.28^A^	0.05	−0.26^A^	0.05	−0.11^B^	0.05	−0.09^C^	0.05	***	−0.17	0.04	−0.200	0.04	Ns
Slope^4^	0.002^ab^	0.004	−0.007^a^	0.004	0.010^b^	0.004	0.008^ab^	0.004	**	0.004	0.001	0.006	0.001	Ns
BW														
Drying (kg)^1^	686^A^	11	695^A^	11	646^B^	11	671^A^	11	***	645^B^	9	704^A^	9	***
Calving (kg)^2^	626^ac^	10	652^b^	10	610^c^	10	639^ab^	10	*	605^B^	9	659^A^	9	***
Difference (kg)^3^	−57.5^A^	7.8	−41.7^AB^	8.2	−35.1^B^	7.9	−27.7^B^	8.1	***	−36.9	7.2	−44.0	7.2	Ns
Slope (kg/day)^4^	−1.13^A^	0.85	−0.38^AB^	0.89	1.38^B^	0.86	0.59^AB^	0.87	***	−0.58^b^	0.76	0.82^a^	0.77	**

Different superscripts for same variables either between production systems or between parity groups denote significant difference: ^a,b^ *P*<0.05; ^A,B^ *P*<0.01.Ns=not significant.

1 As measured on day of dry off.

2 As measured on day of calving.

3 Arithmetical difference between dry off and calving (MJ).

4 Slope of change during the first 15 days of the dry period.

*P<0.05, **P<0.01, ***P<0.001.

### Effects of health group on dry period traits

No significant differences existed between BEC, BCS and BW at drying or calving of cows of different health groups (Table 4). The slope of change in BEC during the first 15 days of the dry period was significantly (*P*<0.05) affected by health group. Cows that developed REPs lost on average −18.26 MJ/day which was significantly different (*P*<0.05) to the rate of change in HC (0.63 MJ/day). The differences in BEC, BCS and BW between drying and calving were significantly different between health groups. Cows that developed REPs lost significantly more (*P*<0.05) BEC, significantly more (*P*<0.001) BCS and significantly more (*P*<0.001) BW than HC. In all cases, no differences existed between cows with metabolic disease and any other diseased group.

**Table 4:** Least squares means and associated SEs of the effect of health status in early lactation on body energy content, body condition score and BW at drying, calving, the rate of change in the first 15 days of the dry period and the arithmetical difference in the traits between drying and calving in Holstein dairy cattle

	Health group								
	Healthy cows		Subclinical mastitis		Reproductive track disorders^1^		Metabolic disorders^2^		
	Mean	SE	Mean	SE	Mean	SE	Mean	SE	*P*
Body energy content									
Drying (MJ)^3^	3059	103	3058	157	3278	133	3144	221	Ns
Calving (MJ)^4^	2817	79	2821	125	2735	105	2573	179	Ns
Difference (MJ)^5^	−235^a^	74	−222^a^	107	−596^b^	101	−422^ab^	171	*
Slope (MJ/day)^6^	0.63^a^	5.11	3.00^ab^	9.60	−18.26^b^	7.44	9.66^ab^	13.95	*
Body condition score									
Drying^3^	2.36	0.045	2.35	0.069	2.46	0.06	2.34	0.1	Ns
Calving^4^	2.24	0.04	2.22	0.06	2.2	0.05	2.1	0.08	Ns
Difference^5^	−0.110^A^	0.036	−0.130^AB^	0.058	−0.270^B^	0.048	−0.240^AB^	0.082	***
Slope^6^	0.039	0.001	0.003	0.001	−0.002	0.001	0.014	0.001	Ns
BW									
Drying^3^ (kg)	667	7.99	666	10.31	674	9.86	693	15.36	Ns
Calving^4^ (kg)	632	7.52	642	10.26	624	9.69	628	16.20	Ns
Difference^5^ (kg)	−35.6^A^	6.1	−20.1^A^	9.6	−55.2^B^	8.1	−51.1^AB^	13.9	***
Slope^6^ (kg/day)	0.59	0.59	0.66	1.11	−1.18	0.87	0.40	1.64	Ns

Different superscripts for same variables between health groups denote significant difference: ^a,b^ *P*<0.05; ^A,B^ *P*<0.01.Ns=not significant.

1 Includes cases of retained placenta and metritis.

2 Includes cases of hypocalcaemia, hypomagnesaemia, ketosis and left displaced abomasum.

3 As measured on day of dry off.

4 As measured on day of calving.

5 Arithmerical difference between dry off and calving (MJ).

6 Slope of change during the first 15 days of the dry period.

*P<0.05, ***P<0.001.

### Effects of production system and parity on dry off traits

Production system had a significant effect on MY at dry off (*P*<0.001). Low forage cows had the highest yield at dry off (23.6 l), which was significantly greater than that of cows from all other systems (Table 5). MBER was significantly greater in low forage cows compared with high forage control cows (*P*<0.05). Parity had no effect on yield at dry off but did have a significant effect on MBER. Cows completing lactation one had greater MBER than those completing lactation two (*P*<0.001).

**Table 5:** Least squares means and associated SEs of the effect of production system and parity on milk yield at dry off and the ratio of milk yield to body energy content on the day of dry off in Holstein dairy cattle

	Production system									Parity at dry off				
	Low forage control^1^		Low forage select^2^		High forage control^1^		High forage select^2^			1		2		
	Mean	SE	Mean	SE	Mean	SE	Mean	SE	*P*	Mean	SE	Mean	SE	*P*
Yield at dry off (l)	23.6^A^	1.1	18.4^BC^	1.1	19.2^B^	1.0	16.3^C^	1.1	***	19.6	0.9	19.2	0.9	Ns
MBER (l/100 MJ)^3^	0.91^a^	0.06	0.81^ab^	0.06	0.68^b^	0.06	0.68^ab^	0.06	*	0.82^A^	0.05	0.73^B^	0.05	***

Different superscripts for same variables either between production systems or between parity groups denote significant difference: ^a,b^ *P*<0.05; ^A,B^ *P*<0.01. Ns=not significant.

1 Control cows were selected to represent average UK genetic merit for milk fat and protein.

2 Select cows were selected to represent the top five per cent of UK genetic merit for milk fat and protein.

3 Ratio of energy-corrected milk yield to body energy content on day of dry off.

*P<0.05, ***P<0.001.

### Effects of health group on dry off traits

Yield at dry off was significantly different between cows belonging to different health groups (*P*<0.05) (Table 6). Of the cows that developed disease in the first 30 days of lactation, those that developed SCM had significantly higher yields (21.3 l/day) than those that developed REPs or metabolic disease. Average dry off yield of HC was not significantly different from the dry off yield of any other health group. Cows that developed SCM in the first 30 days of lactation had a significantly higher MBER (0.92) than HC and those that developed REPs (*P*<0.05).

**Table 6:** Least squares means and associated SEs of the effect of health status in early lactation on milk yield at dry off and the ratio of milk yield to body energy content on the day of dry off in Holstein dairy cattle

	Health group								
	Healthy cows		Subclinical mastitis		Reproductive track disorders^1^		Metabolic disorders^2^		
	Mean	SE	Mean	SE	Mean	SE	Mean	SE	*P*
Yield at dry off (l)	19.9^ab^	0.78	21.3^b^	1.08	18.6^a^	1.00	17.8^a^	1.69	*
MBER^3^ (l/100 MJ)	0.81^a^	0.044	0.92^b^	0.059	0.74^a^	0.055	0.70^ab^	0.093	*

Different superscripts for same variables between health groups denote significant difference: ^a,b^ *P*<0.05; ^A,B^ *P*<0.01.

1 Includes cases of retained placenta and metritis.

2 Includes cases of hypocalcaemia, hypomagnesaemia, ketosis and left displaced abomasum.

3 Ratio of energy-corrected milk yield to body energy content on day of dry off.

*P<0.05.

## Discussion

This study has demonstrated that cows which develop different production diseases in early lactation exhibit different physiological and production characteristics during the changeover and dry periods. Measurable differences in physiology and production can be exploited in such a way as to extract indicators of a risk of future disease. In the current analysis, dry off yield, MBER, the rate of change in BEC during the first 15 days of the dry period and the difference in BW, condition and energy content across the dry period were significantly different between HC and those that develop post-calving production disease. Therefore, these traits are potential disease indicators. MBER and yield at dry off are significantly higher in cows which go on to develop early lactation SCM; this suggests that these traits may be useful as indicators of early lactation SCM from as early as the end of the previous lactation. The rate of change in BEC during the first 15 days of the dry period – ‘the changeover period’ – is significantly different between cows that developed REPs post-calving and cows that did not develop clinical disease. Similarly, loss in BW, BCS and BEC from dry off to calving could be used to identify cows which go on to develop post-calving reproductive track conditions. Cows which went on to develop REPs lost significantly more BW, condition and energy content than HC. The current study highlights that on average different loss patterns are experienced at critical time points in the lactation–gestation cycle between cows that go on to develop different diseases.

The energy status of the cow during the dry and transition periods is critical in determining the success of the lactation–gestation cycle. During this time, late-term foetal growth, parturition and the initiation of lactation are accompanied by significant endocrine changes which are in excess of those occurring at any other stage in the dairy cows’ production cycle (Grummer *et al*., 2004). The sudden increase in nutrient demands required to facilitate these physiological tasks, coupled with suppressed dietary intake potential, results in a state of NEB (Frigo *et al*., 2010). During late-term pregnancy, the cows’ priority is to prepare for the next lactation; hence her strategy is to build up body reserves. Rapid mobilisation of these reserves post-calving facilitates milk production and allows the cow to reach optimal condition for re-breeding. These sequential priorities and strategies mean that the cow transitions through a cyclic and genetically driven pattern of lipid reserves (Friggens *et al*., 2007). It is critical that the cow is supported, through optimum feeding and management, in order that she is allowed to follow this natural cycle of body reserve mobilisation and accretion. Disruptions to this cycle not only can have negative consequences for the offspring and productivity but those that cause extended of more severe periods of NEB have been shown to be linked with increased levels of metabolic and production disease in early lactation (Roche and Berry, 2006).

In the current study, cows with a high dry off yield developed early lactation SCM. It has previously been reported that cows which have not had a significant reduction in MY before dry off have higher levels of intramammary infection compared with cows whose daily yield had reduced in the period before dry off, although the optimal level of production at dry off is not clear (Dingwell *et al*., 2001). The majority of epidemiological studies of mastitis, including that of Dingwell *et al*. (2001) focus mainly on dry period acquired infections however, in the current study, no distinction was made between cases of persistent infection and cases of dry period acquired infection. Similar to MY, cows with a high MBER developed early lactation SCM. In theory, this may indicate that cows with a high MBER value would benefit from a shortened dry period, whereas those with a low MBER value would benefit from an extended dry period in order to relieve them of the energy demands for milk production and to allow them to modulate their body reserves in preparation for parturition and the following lactation (Friggens *et al*., 2004). Further research to address the effect of shortened and extended dry period lengths on physiology and production are necessary.

Relative to HC, animals that lost condition at the highest rate during the first 15 days of the dry period went on to develop REPs in the *postpartum* period. Cows that developed retained placenta or metritis lost, on average, 18.26 MJ of BEC per day for this 15-day period, whereas cows which did not develop clinical disease gained an average of 0.63 MJ/day. Garnsworthy (2006) argued that the rate of mobilisation of body reserves may be of greater importance in managing the risk of disease in the transition period than over-conditioning, as had been previously been thought. Rapid mobilisation of reserves causes physiological stress which manifests itself in suppressed DM intake and MY in early lactation alongside an increased incidence of health and reproductive problems (Roche and Berry, 2006). This may explain the biology which underpins the results obtained in this study; that cows that developed REPs post-calving experienced rapid mobilisation of body reserves in the early dry period. Similarly, Kim and Suh (2003) found that cows that experienced a marked loss in condition over the dry period (1 to 1.5 point loss) took longer to regain condition post-calving than those that experienced only a moderate loss in condition (0 to 0.75 point loss). Incidence of metritis and metabolic diseases was significantly greater amongst the cows that had lost between 1 and 1.5 points of BCS, compared with those who lost between 0 and 0.75. Cows which experienced rapid loss of condition in the changeover period went on to develop retained placenta and metritis after calving. This finding highlights the importance of the far-off dry period and the relevance of studying the whole dry period when considering disease risk in the following lactation. As such, attention should not focus solely on the transition period. The early stage of the dry period is important as cows undergo significant physiological change as they change from lactating to dry cows.

In addition, the rate of change in BEC in the changeover period was significantly affected by production system. Irrespective of genetic merit, cows fed a low forage diet mobilised reserves throughout this period, whereas those fed a high forage diet accreted reserves. This may be explained by the significantly greater BEC of cows fed a low forage diet at dry off, compared with those fed a high forage diet. Garnsworthy and Topps (1982) demonstrated that cows with a higher BCS at calving lost more BW and condition in early lactation than cows of modest condition at calving. This relationship was further investigated by Broster and Broster (1998), who indicated that over-conditioned cows were shown to experience more rapid mobilisation of body energy reserves in early lactation than those in optimum condition. In the current study, higher BEC at drying appears to be associated with a greater loss in condition in the early dry period.

Cows that developed REPs immediately post-calving lost more than double the amount of BCS and BEC in the preceding dry period than cows that that did not develop clinical disease. In terms of BW, cows that developed REPs lost 55% more BW than the cows which did not develop any disease in the early lactation period. Given that there is no significant difference in BW, BCS or BEC of cows with and without disease at dry off and calving, it would seem that it is the change in these traits during the dry period that exert an influence on future disease risk rather than the absolute level of each of the traits. The fact that no difference exists between healthy and diseased cows in BCS at dry off and calving supports the theory of Garnsworthy and Topps (1982) that all cows strive to achieve similar body energy targets at critical points in the lactation–gestation cycle.

During lactation, cows can be forced from their natural body energy cycle by environmental factors specific to the lactation period. In the dry period when milk production ceases and management is less intensive, cows are offered the opportunity to modulate their body energy reserves according to their genetic predispositions. However, in previous studies weight loss in the dry period has been associated with increased mortality and *postpartum* complications and even moderate levels of fat mobilisation can induce NEB and have an adverse effect on health (Gearheart *et al*., 1990). In their study Gearheart *et al*. (1990) found that cows that lost the most condition in the dry period developed dystocia or were culled in the subsequent lactation. It would be logical to assume that these cows were over-conditioned at dry off and therefore were mobilising reserves in order to reach optimal calving condition. However, similar to the results in the current study, cows which lost the most condition over the dry period were assessed to be of the same body condition as HC at dry off (Gearheart *et al*., 1990). Markusfeld *et al*. (1997) report similar results; cows that lost most condition during the dry period had an increased incidence of retained placenta and metritis. The mean loss of condition incurred by multiparous cows was 0.33 BCS units. In contrast to the work of Gearheart *et al*. (1990) and to this study, Markusfeld *et al*. (1997) additionally reported a significant relationship between BCS at drying off and condition change in the dry period. The heaviest cows at dry off lost more weight during the dry period.

Although no significant differences existed in absolute BW, BCS and energy content at dry off between healthy and diseased cows in this study, their importance cannot be entirely dismissed. Ranges between the minimum and maximum BCS, in the current study, were small and therefore the power to assess the effect of true over and under-conditioning was limited. The findings of this study may have differed under different herd size, management or feed systems. However, although data used in this study was sourced from one farm, the four dairy production systems in operation throughout the course of this study represented contrasting approaches to dairy herd management and reflected a range of possible dairy systems. Inclusion of production system in the analyses allowed the effect of genotype and environment to be accounted for in addition to other factors. Further, the rich longitudinal nature of the database afforded the opportunity to access BW and BCS data for individual cows over an extended period of time, throughout which all aspects of management and production were recorded.

## Conclusion

This study has demonstrated that cows which developed different diseases in the first 30 days of lactation had different characteristics in their physiology and production traits during the changeover and dry periods. The changeover from the previous lactation to the dry period has been identified as a critical time in the lactation cycle. Thus, the changeover period requires careful management so as to avoid rapid mobilisation of body energy reserves which have been associated with increased risk of disease in the following lactation. It has been generally accepted that nutritional management in the dry period affects the metabolic status of the cow in the subsequent lactation (Andersen *et al*., 2008). However, monitoring should not be limited to the dry period. It should rather be a continuous process including the changeover period between lactations. The results from the current study have important implications for the inclusion of on-farm data in models for the prediction of disease risk. Further, this analysis contributes to the development of precision farming tools which may utilise routinely recorded farm data. Overall, this study lays the foundation for the increased use of data which is easily recordable on-farm to be used in disease risk calculation.
